# Optical Ising machine

**DOI:** 10.1093/nsr/nwag261

**Published:** 2026-05-12

**Authors:** Ming Li, Yanbo Ren

**Affiliations:** State Key Laboratory of Optoelectronic Materials and Devices, Institute of Semiconductors, Chinese Academy of Sciences, China; Center of Materials Science and Optoelectronics Engineering, University of Chinese Academy of Sciences, China; School of Electronic, Electrical and Communication Engineering, University of Chinese Academy of Sciences, China; State Key Laboratory of Optoelectronic Materials and Devices, Institute of Semiconductors, Chinese Academy of Sciences, China; Center of Materials Science and Optoelectronics Engineering, University of Chinese Academy of Sciences, China; School of Electronic, Electrical and Communication Engineering, University of Chinese Academy of Sciences, China

## Abstract

Optical Ising machines harness physical dynamics for combinatorial optimization, with integrated photonics enabling scalable, high-speed, energy-efficient solutions for real-world applications.

The Ising machine constitutes a fundamentally new computing paradigm purpose-built for combinatorial optimization problems (COPs). In contrast to the memory–processor separation in von Neumann architectures, it draws inspiration from the Ising model in statistical physics and computes through the intrinsic energy-minimization dynamics of physical systems. Binary spins that take values of +1 or −1 serve as computational primitives. By mathematically mapping the integer variables of a COP onto one or more spins, the constraints of the COP are transformed into the spin–spin coupling coefficients of the Ising model, rendering the solution of the COP equivalent to finding the ground state of the corresponding Ising problem [1,2]. It has been mathematically proven that many classical COPs can be cast into the Ising form [3]. By encoding the spin–spin coupling coefficients associated with the target COP into an Ising machine, the system naturally relaxes toward its lowest-energy configuration (the ground state), which directly corresponds to the optimal solution of the original problem. This physics-based computing architecture differs fundamentally from conventional digital computers designed for general-purpose computation, as it is a specialized computing architecture dedicated to solving COPs. Such a task-specific design enables Ising machines to potentially achieve faster solution times for COPs compared with conventional digital computers [2]. As shown in Fig. 1, as practical computing architectures for real-world COPs, Ising machines are evaluated by several key metrics, including spin scalability, coupling precision, system stability, and energy efficiency. Spin scalability and the coupling precision determine the accessible problem size and complexity. System stability governs solution fidelity and long-term operational reliability. Meanwhile, energy efficiency directly impacts the economic cost of computation and ultimately shapes the feasibility of large-scale deployment.

**Figure 1.**
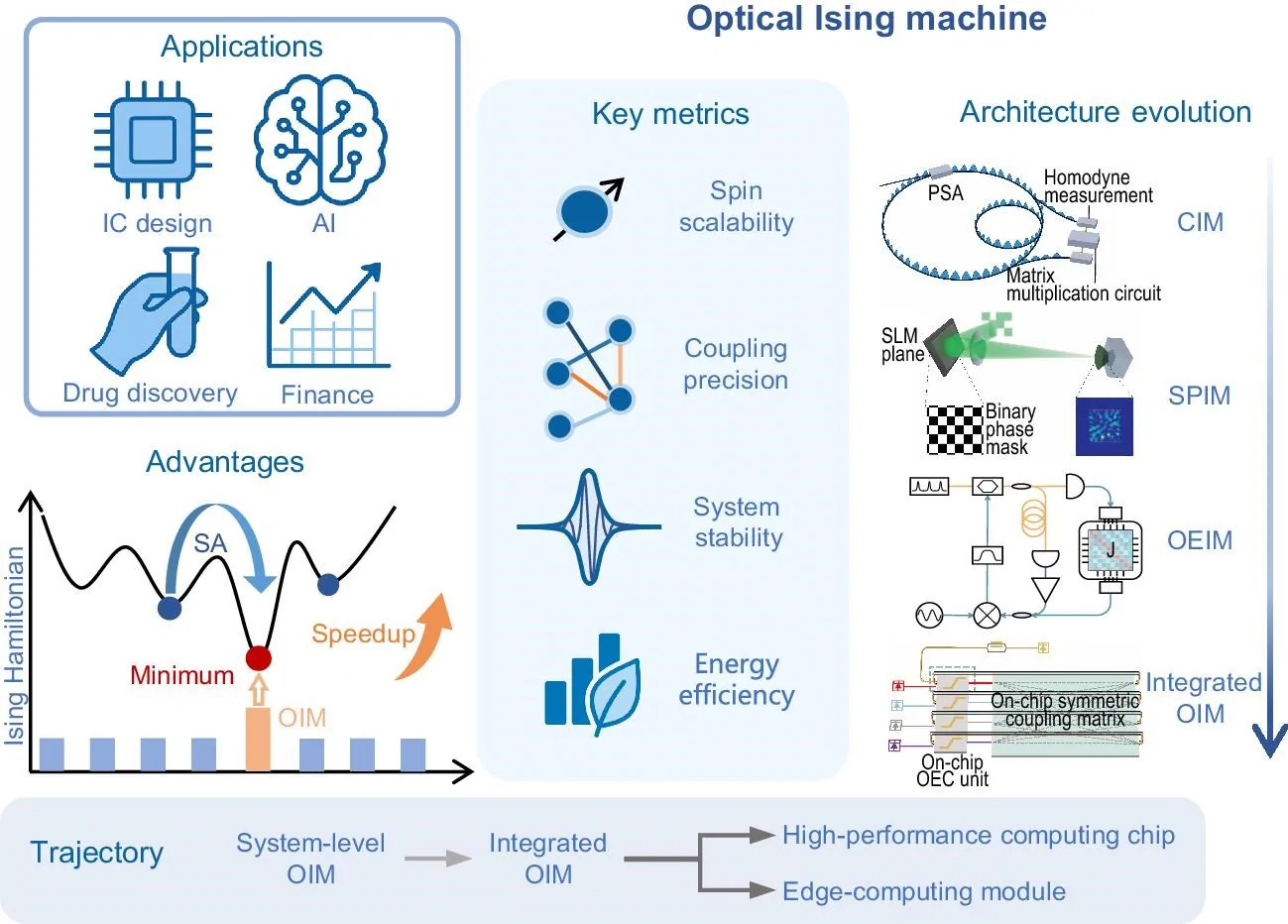
Optical Ising machine (OIM). OIMs are high-efficiency computing architectures tailored for combinatorial optimization problems in fields such as drug discovery and artificial intelligence. By exploiting the natural dynamics of physical systems, OIMs have the potential to achieve faster solution times than simulated annealing (SA) implemented on conventional digital computers for certain combinatorial optimization tasks. Key metrics of OIMs include spin scalability, coupling precision, system stability, and energy efficiency. To advance these metrics, several implementations have been developed, including coherent Ising machines (CIMs) [2], spatial photonic Ising machines (SPIMs) [5], optoelectronic Ising machines (OEIMs) [6], and integrated OIMs [9]. The overall technological trajectory is a transition from system-level platforms toward fully integrated architectures, which are expected to further evolve into high-performance computing chips and edge-computing modules, thereby greatly enhancing their practical value.

Ising machines have been realized on a range of physical platforms, including electronic circuits, optical systems, and quantum systems. Among these, optical Ising machines (OIMs)—powered by optical components and physical principles—offer distinct advantages in key metrics and have rapidly become a central research focus. System-scale OIMs constructed from discrete components have demonstrated faster solution times than conventional digital computers for certain benchmark optimization problems, and the technology is now advancing toward chip-scale optoelectronic integration. Representative OIM architectures at the system level include the coherent Ising machine (CIM), the spatial-photonic Ising machine (SPIM), and the optoelectronic Ising machine (OEIM). The CIM, the earliest optical realization of the Ising machine, employs networks of degenerate optical parametric oscillators whose minimum-dissipation dynamics drive the system toward the ground state, enabling efficient solutions to COP instances with up to 100 000 variables [4]. The SPIM, combining spatial light modulators with wavelength-division multiplexing, further boosts computational parallelism and energy efficiency [5]. The OEIM integrates optical and microwave technologies, extending stable operation from seconds to hours and achieving coupling precision as high as 16 bits [6]. To reduce cost and energy consumption, improve system stability, and enable practical deployment, researchers are developing integrated OIMs based on advanced photonic integrated circuit technologies. In typical implementations, on-chip Mach–Zehnder interferometers realize matrix–vector multiplication, while electrical or optical nonlinear modules implement the spin-update dynamics [7–9]. Integrated OIMs with tens to thousands of spins have demonstrated the feasibility of this integrated approach.

OIMs offer several advantages over both conventional digital computers and quantum annealers for solving COPs. OIMs solve COPs by exploiting the intrinsic self-convergence and high parallelism of optical or optoelectronic systems; digital computers rely on transistor-based logic circuits to execute optimization algorithms; and quantum annealers leverage quantum tunneling and adiabatic evolution to perform the optimization. Benefiting from intrinsic physical parallelism, OIMs can solve classical benchmark problems such as Max-Cut faster than digital computers while achieving comparable and practically acceptable solution quality. However, given a sufficiently long runtime, advanced optimization algorithms on digital computers can still obtain solutions of higher quality [4]. Using photons as information carriers also removes resistive and thermal dissipation, drastically lowering the energy required per computation. In contrast to quantum annealers, OIMs can be scaled more straightforwardly to architectures comprising hundreds of thousands of spins, while operating stably at room temperature without cryogenic refrigeration or magnetic shielding. In terms of computational speed, previous studies indicate that quantum annealers outperform OIMs for Max-Cut problems on sparse graphs. However, for dense Max-Cut instances, OIMs that realize all-to-all connectivity via spatial light modulators or field-programmable gate arrays can exhibit better performance [10]. OIMs are rapidly advancing toward practical deployment. In urban traffic optimization, OIMs have reduced computation time from minutes to milliseconds, enabling real-time traffic optimization [6]. In communication networks, OIMs have been applied to routing and resource allocation, improving overall throughput and network capacity. Research and industrial efforts are increasingly converging. In the United States and Japan, large-scale CIM systems with more than 100 000 spins have been demonstrated and applied to airline scheduling and logistics optimization, alongside active exploration of chip-scale nonlinear platforms [11,12]. European groups are driving progress in SPIM

architectures, achieving further gains in energy efficiency and solution quality through hybrid integration of classical algorithms with optical computing systems [13]. In China, an application-oriented strategy is being pursued, emphasizing improvements in system-level OIM performance while simultaneously accelerating the development of integrated OIM technologies [6,7].

System-level OIMs have already demonstrated notable performance advantages and clear prospects for commercialization. However, to date, system-level OIMs have not yet exhibited transformative advantages over conventional algorithms in practical applications, and their performance metrics indicate considerable scope for further improvement. Accordingly, their further development will rely on continued innovation in supporting algorithms and system architectures, such as evolution-dynamics-based spin error-correction algorithms to enhance solution quality [14], as well as architectural optimizations to expand spin scalability and improve coupling precision, system stability, and energy efficiency, thereby strengthening their practical utility. At the same time, the development of application-specific Ising computing frameworks is essential to enable deeper integration with real-world industrial requirements.

Integrated OIMs are still in their formative stage and require substantial advances across several key metrics. Even so, the analog and intrinsically parallel nature of the Ising computing paradigm aligns exceptionally well with the strengths of optoelectronic integration, positioning OIMs as a defining and highly promising application of integrated photonics [15]. By eliminating the reliance on hundreds of meters of optical fiber and numerous discrete active components, integrated OIMs can sharply reduce both energy consumption and fabrication cost, thereby enabling standardized, high-volume manufacturing. Moreover, integrated OIMs can function as modular computational accelerators that interface seamlessly with on-chip computing, communication, and sensing subsystems, supporting compact, efficient, and scalable co-processing architectures.

At present, integrated OIMs face two principal technological challenges: realizing strong on-chip nonlinearities and scaling to large spin counts. Nonlinearity is indispensable for driving spin evolution and ensuring convergence of the system. Yet the Kerr nonlinearity of silicon (4.5 × 10^−18^ m²/W) is far too weak to sustain the required nonlinear processes. Current solutions rely on heterogeneous integration of highly nonlinear materials, such as thin-film lithium niobate, or on exploiting electronic-domain nonlinearities within optoelectronic hybrid circuits [7,9]. While effective, these approaches incur additional insertion loss, increase fabrication complexity and cost, and can introduce thermal or noise-related instabilities that degrade computational fidelity. Looking ahead, silicon-compatible nonlinear enhancement mechanisms—such as carrier-induced dispersion and optomechanical interactions—offer an attractive alternative, as they avoid heterogeneous integration while providing low loss and high manufacturability. The scalability of integrated OIMs is further limited by on-chip optical loss. Although silicon photonic waveguides have achieved propagation losses as low as ∼1 dB/cm at the 1550 nm band, the silicon microring modulators and Mach–Zehnder interferometers required for spin readout and update still introduce non-negligible insertion loss. Consequently, the cumulative optical attenuation grows rapidly with spin count, limiting present silicon-based integrated OIMs to at most a few hundred spins [8,9]. To overcome these limitations, researchers are pursuing wavelength-division multiplexing to expand spin count in the frequency domain, as well as hybrid optoelectronic architectures that employ electronic registers for storing and accessing large-scale spin states. Recently, a 256-spin fully connected integrated OIM has been demonstrated on a lithium-niobate-based optoelectronic integrated chip [16]. This work explores the feasibility of constructing large-scale, practically viable integrated OIMs and represents a milestone in the development of integrated OIM platforms.

As shown in Fig. 1, looking ahead, integrated OIMs are expected to evolve along two converging trajectories. The first focuses on extreme computational performance: leveraging advanced photonic–electronic integration to push key metrics to their limits, developing dedicated high-performance computing chips, and assembling multi-chip clusters to achieve large-scale computational acceleration. Such systems could offer overwhelming advantages in both speed and energy for tasks such as cryptographic decoding and protein folding prediction. In AI, integrated OIMs can operate as high-efficiency physical samplers to accelerate inference in large-scale machine-learning models, and as hardware realizations of restricted Boltzmann machines that enable contrastive-divergence training without backpropagation. Such physics-driven dynamics inherently avoid the gradient-vanishing and gradient-exploding problems that afflict conventional deep neural networks.

The second trajectory targets deep integration with real-time application scenarios. In this direction, integrated OIMs operate as edge-computing modules that are tightly coupled to on-chip photonic sensing front-ends (e.g. light detection and ranging, LiDAR). Streaming sensory data directly drives the optimization process and decision feedback, forming an integrated sensing-and-computing closed-loop system. In autonomous driving, such real-time coordination between sensors and integrated OIMs can enable rapid path planning and obstacle avoidance, thereby reducing decision latency. For Unmanned Aerial Vehicle swarms or coordinated industrial robots, complex task allocation and trajectory planning can be executed in real time, enabling efficient collective behavior. This perception-guided decision-making paradigm has the potential to overcome the high-latency sense-transmit-cloud-compute-actuate pipeline, making it particularly advantageous for latency-critical optimization and control tasks.
